# Nucleic acid therapeutics as differentiation agents for myeloid leukemias

**DOI:** 10.1038/s41375-024-02191-0

**Published:** 2024-02-29

**Authors:** Olivia Kovecses, François E. Mercier, Maureen McKeague

**Affiliations:** 1 Department of Pharmacology and Therapeutics, McGill University, Montreal, H3G 1Y6 QC Canada; 2 Division of Hematology and Experimental Medicine, Department of Medicine, McGill University, Montreal, H3T 1E2 QC Canada; 3 Department of Chemistry, McGill University, Montreal, H3A 0B8 QC Canada

**Keywords:** Drug development, Haematological cancer, Leukaemia

## Abstract

Differentiation therapy has proven to be a success story for patients with acute promyelocytic leukemia. However, the remaining subtypes of acute myeloid leukemia (AML) are treated with cytotoxic chemotherapies that have limited efficacy and a high likelihood of resistance. As differentiation arrest is a hallmark of AML, there is increased interest in developing differentiation-inducing agents to enhance disease-free survival. Here, we provide a comprehensive review of current reports and future avenues of nucleic acid therapeutics for AML, focusing on the use of targeted nucleic acid drugs to promote differentiation. Specifically, we compare and discuss the precision of small interfering RNA, small activating RNA, antisense oligonucleotides, and aptamers to modulate gene expression patterns that drive leukemic cell differentiation. We delve into preclinical and clinical studies that demonstrate the efficacy of nucleic acid-based differentiation therapies to induce leukemic cell maturation and reduce disease burden. By directly influencing the expression of key genes involved in myeloid maturation, nucleic acid therapeutics hold the potential to induce the differentiation of leukemic cells towards a more mature and less aggressive phenotype. Furthermore, we discuss the most critical challenges associated with developing nucleic acid therapeutics for myeloid malignancies. By introducing the progress in the field and identifying future opportunities, we aim to highlight the power of nucleic acid therapeutics in reshaping the landscape of myeloid leukemia treatment.

## Introduction

Acute myeloid leukemia (AML) develops due to the inefficient differentiation and uncontrolled proliferation of immature myeloblasts. As all AML subtypes result from a differentiation block, the differentiation process is a possible therapeutic target. In acute promyelocytic leukemia (APL) for example, the combination of all-trans retinoic acid (ATRA) and arsenic trioxide has transformed APL from a deadly diagnosis into a highly curable disease. ATRA acts to restore the expression of pro-differentiation genes that are transcriptionally repressed by the fusion oncoprotein PML::RARɑ and together with arsenic trioxide also results in proteosome-mediated degradation of PML::RARɑ [1]. More recently, hypomethylating agents and certain targeted therapies are reported to exert their therapeutic effect by inducing terminal differentiation [2]. Yet, outside of APL, differentiation agents are not used with curative intent.

All leukemic blasts originate from a relatively small population of leukemic stem cells (LSCs) which evolved during clonal hematopoiesis. Due to their quiescent state and unique metabolism, LSCs evade the eradicating effects of cytotoxic agents that target rapidly dividing cells, resulting in eventual relapse in many patients [3]. Therefore, a lasting cure for AML depends on eliminating LSCs and reducing leukemic blast burden. Differentiation therapy has the potential to improve patient outcomes in AML due to its unique approach of inducing leukemic blasts, and even LSCs, to mature into functional, non-malignant cells [4]. This strategy is targeted and less toxic as it spares healthy cells and reduces side effects associated with the widespread damage caused by cytotoxic chemotherapies.

Myeloid differentiation is primarily regulated by transcription factor networks, non-coding RNAs (ncRNA), and signaling cascades that dictate lineage commitment decisions [5] and are communicated via protein-protein, protein-DNA, RNA-DNA, or RNA-RNA interactions. As these interactions often lack well-defined ligand binding sites [6] and occur in difficult-to-access nuclear locations [7], it remains a challenge to develop small molecule inhibitors capable of effectively modulating myeloid differentiation. Nucleic acid therapeutics (NATs) are poised to address these challenges given that they can target genes and transcripts based on sequence, circumventing “undruggable” interactions and targets lacking binding pockets. Specifically, NATs utilize DNA or RNA molecules to modulate gene expression or protein function [8], offering a personalized treatment based on a patient’s genetic profile.

The goal of this review is to promote collaboration between the fields of oligonucleotide chemistry and hematology-oncology to advance the development of differentiation therapies for devastating myeloid malignancies. We describe key mechanisms and applications of NATs, review pre-clinical and clinical examples of NATs as differentiation agents in myeloid leukemias, and provide NAT design and delivery strategies to modulate relevant AML therapeutic targets.

## Mechanism and molecular design of nucleic acid therapeutics

Here, for each NAT modality, we describe the mechanism and current approval phase to provide context for the applicability of NATs for myeloid leukemias.

### Small interfering RNA

**Fig. 1:**
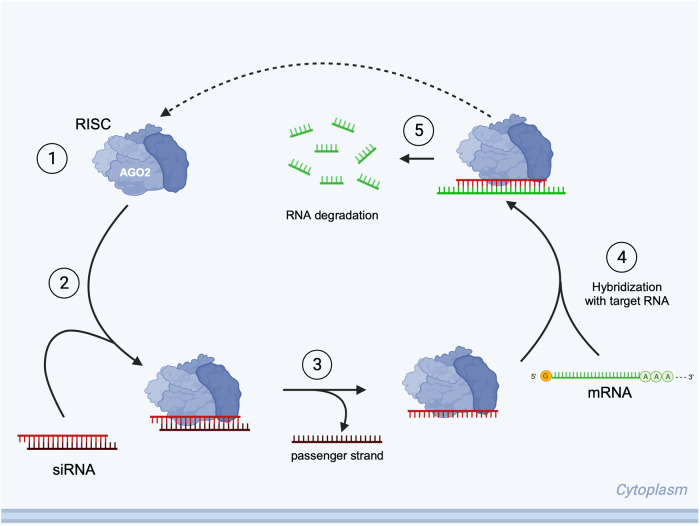
Mechanism of small interfering RNA. RNAi is executed by the RNA-Induced Silencing Complex (RISC), a multiprotein complex with Argonaute-2 (AGO2) as the main effector protein. Following delivery of siRNA to the cytoplasm and assembly of the RISC (step 1), the short RNA duplex is loaded onto RISC (step 2) and the strands are separated (step 3), with the most energetically favorable strand being incorporated into the complex. The RISC then associates with target messenger RNA (mRNA) via complementary binding to the single-stranded siRNA (step 4), and the catalytic site of AGO2 cleaves the mRNA into small fragments (step 5). Effective mRNA degradation via RNAi can silence gene expression below detectable levels.

Small interfering RNAs (siRNAs) are short RNA duplexes that take advantage of the RNA interference (RNAi) pathway discovered by Fire & Mello [9], thereby allowing for exogenous control over gene expression (Fig. 1) [10]. Since the approval of the first siRNA drug Patisiran [11] in 2018, five other siRNA therapies received approval from the Food and Drug Administration (FDA) for the treatment of genetic disorders. There are eight siRNA formulations in phase I-II clinical trials for cancers, including B-cell non-Hodgkin’s lymphoma (NCT04995536); however, there are currently no siRNAs approved or in clinical trials for myeloid leukemias.

siRNAs provide precise silencing of mutated or overexpressed transcripts due to their ability to differentiate target RNAs from the rest of the transcriptome. Therefore, siRNAs can selectively target fusion transcripts from chromosomal rearrangements, genes upregulated due to epigenetic activation, and ncRNA that act to suppress expression of anti-proliferative genes in leukemias.

### Antisense oligonucleotides

**Fig. 2:**
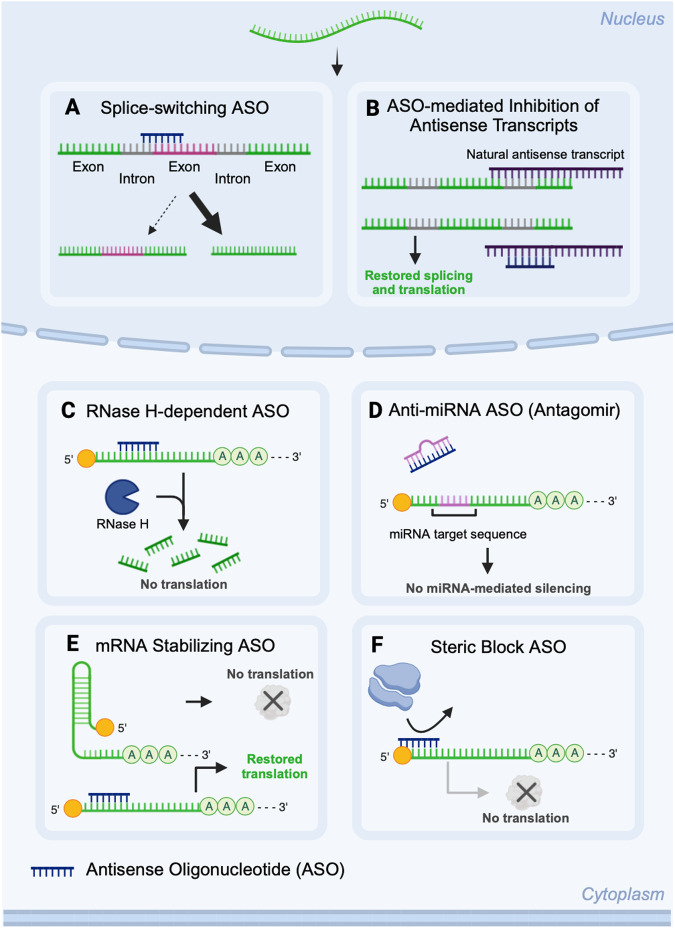
Mechanism of antisense oligonucleotides. **A** Splice-switching ASOs interfere with RNA-binding proteins and non-coding RNA that direct splicing. By preventing binding of splicing machinery to the splice site or blocking pro-splicing effects, ASOs enable entire exons to be excluded from mature mRNA. Alternatively, by blocking binding of splicing repressors, ASOs ensure inclusion of target exons in mature mRNA. **B** ASO-mediated inhibition of natural antisense transcripts regulates antisense transcripts produced from the antisense strand of DNA during transcription of the protein-coding gene located on the sense strand. Antisense transcriptions negatively or positively regulate the expression of their corresponding sense gene either by interacting directly with pre-mRNA or by regulation transcription of the gene itself. **C** RNase H1-dependent ASOs hybridize to complementary target RNA forming a DNA-RNA heteroduplex that results in RNase H1 cleavage of the RNA. **D** Anti-miRNA ASOs (antagomirs) sequester mature miRNA through complementary binding to their seed region (nucleotides 2 to 8 of miRNA). Such ASOs competitively inhibit the endogenous function of a target miRNA and block the ability of miRNA from either inhibiting or enhancing gene expression. **E** mRNA stabilizing ASOs promote mRNA stability by preventing formation of 5’-UTR secondary structures; or redirecting polyadenylation to an alternate upstream site to remove 3’-UTR destabilizing regions. **F** Steric block ASOs physically block translational machinery to the target mRNA. In mRNAs with multiple start codons, steric block ASOs redirect translational machinery to a secondary start codon, thereby allowing selective expression of specific protein isoforms.

Antisense oligonucleotides (ASOs) are short single-stranded oligonucleotides that target and alter RNA processing, thereby regulating translation. ASOs are mechanistically unique due to their ability to either silence, enhance, or manipulate the expression of gene variants [12]. Six distinct ASO mechanisms have been identified: RNase H1-dependent ASOs and several RNase H1-independent mechanisms [13, 14], which include steric block ASOs, splice-switching ASOs, antagomir ASOs (anti-miRNA), mRNA stabilizing ASOs, and ASO-mediated inhibition of antisense transcripts (Fig. 2).

Over the past 25 years, ten ASO drugs have obtained FDA-approval, yet there are currently no ASOs approved for the treatment of any myeloid malignancy. As we will describe later, clinical trial recruitment is in progress for two steric block ASO candidates for AML: a phase IIb trial of “BP1001” in combination with venetoclax/decitabine for newly diagnosed AML (NCT02781883), and a phase I/Ib study of “BP1002” for refractory/relapsed AML (NCT05190471). Unfortunately, three ASOs failed in clinical trials for AML. While all were well tolerated by patients (NCT00780052, NCT00085124, NCT01018069), complete remission was sub-optimal.

Despite certain setbacks, ASOs are a promising and applicable therapeutic strategy for myeloid leukemias because the range and variety of their therapeutic mechanism matches the genetic diversity and heterogeneity found in myeloid leukemias. For example, antagomirs correct miRNA imbalances, while steric block ASOs reduce the cellular burden of oncogenic genes. Furthermore, different ASOs could be mixed and combined to target the spectrum of genetic dysregulation.

### Aptamers

**Fig. 3:**
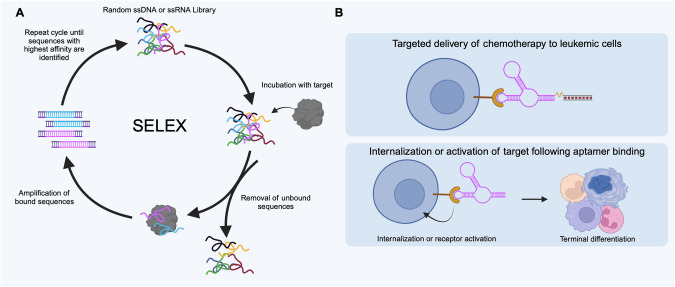
Aptamers as therapeutics and delivery tools. **A** The SELEX process involves iterative rounds of incubating the target of interest with large oligonucleotide libraries, washing, and collecting the highest binding sequences. **B** Therapeutic aptamers can function as agonists or antagonists. As agonists, they induce an active conformation of the receptor through allosteric interactions. As antagonists, they physically block the binding site of the receptor, induce inactive conformations through allosteric binding, interfere with receptor dimerization, reduce cell surface receptors by initiating internalization or degradation, and/or inhibit signal transmission by binding to a messenger molecule. Aptamer-based drug delivery strategies help deliver therapeutic agents to the target site via conjugation to a specific agent minimizing off-target effects.

Aptamers are single-stranded oligonucleotides that fold into 3D structures enabling specific binding to various targets. These molecules cannot be designed via complementarity but must be selected via an integrative in vitro selection process known as Systematic Evolution of Ligands by Exponential enrichment (SELEX) (Fig. 3A). Aptamers function much like antibodies, but are more easily chemically synthesized, highly stable, and reversible in vivo [15]. As such, aptamers can act as therapeutics and drug delivery tools [16] (Fig. 3B).

Two aptamer therapies are on the market, the first was approved in 2004 (Pegaptanib) and very recently Avacincaptad pegol (2023) received FDA approval, though both for age-related macular degeneration. One aptamer candidate, AS1411, has been tested for relapsed/refractory AML in a phase II clinical trial [17]. The study reported an optimistic outcome for patients receiving the combination of AS1411 with high dose cytarabine, but no follow up study has been conducted since the 2009 report. Regardless, there is interest in aptamers for various cancers as evidenced by Olaptesed pegol, an aptamer candidate in clinical trials for pancreatic cancer (NCT04901741). However, perhaps where aptamers are poised for the most important impact is in their targeted delivery. Aptamers can target cell surface markers and interfere with oncogenic signalling by either inhibiting or activating the receptor. Given that leukemic blasts and LSCs can be distinguished from healthy cells via cell surface markers, patient-tailored aptamers may offer improved drug-selectively to target challenging LSC populations buried in the bone marrow niche.

### Small activating RNA

**Fig. 4:**
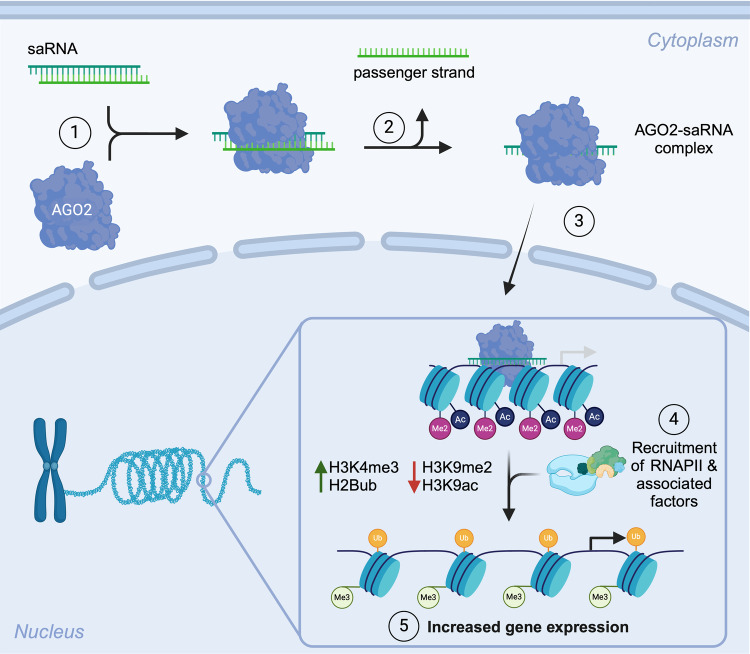
Mechanism of small activating RNAs. saRNAs reversibly increase mRNA expression above endogenous levels by targeting the promoter region of the gene of interest. Once the saRNA is delivered to the cytoplasm, AGO2 and heterogeneous nuclear ribonucleoproteins (hnRNPs) bind to the saRNA duplex. The guide strand is retained, and the passenger strand is discarded. Following translocation of saRNA-AGO2/hnRNPs complex to the nucleus, the guide strand will bind to a complementary sequence near the promoter, resulting in recruitment of transcription initiation and elongation factors, such as RNA polymerase II, RNA helicase A, RNA polymerase-associated protein CTR9 homolog, and RNA polymerase II-associated factor 1 homolog. At the nucleosome level, saRNAs activate transcription by loosening the chromatin through epigenetic changes and histone modifications, such as reduced acetylation and dimethylation of histone H3K9, increased di/trimethylation at histone H3K4, and monoubiquitination on histone H2B [18]. These epigenetic changes are potentially responsible for the long-lasting and sustained gene upregulation induced by saRNAs.

Small activating RNAs (saRNAs) target genomic loci and transcriptionally activate gene expression (Fig. 4) [18]. saRNAs are the most recent NAT, and therefore most designs are in the pre-clinical stages of development, and none have received FDA-approval. However, one saRNA candidate, MTL-CEBPA, is currently in phase II clinical trials for hepatocellular carcinoma (NCT04710641) [19].

There are currently no saRNAs approved or in clinical trials for myeloid leukemias. Interest in these NATs is increasing given that many transcription factors responsible for myeloid differentiation are epigenetically silenced or inhibited. Given pharmacological challenges to restore their expression, saRNAs can increase the repertoire of drug targets in myeloid leukemias by restoring target activity to “healthy” levels.

## Challenges with developing nucleic acid therapeutics for myeloid leukemias

Significant progress has been made, particularly by chemists, to transform nucleic acids into drug-like compounds. As of January 2024, 17 NATs are FDA approved [20], and many more are in clinical trials. Nonetheless, several challenges remain. Here, we describe some of the general challenges in developing NATs and unique challenges in NAT development for myeloid malignancies.

### Universal challenges with developing NATs

There are universal challenges with developing NATs independent of the target disease. It is relatively “straightforward” to design and screen a large array of NATs for a specific target (often hundreds or more sequences). However, translating “hits” from the discovery phase to preclinical and then clinical phase is not trivial in part because chemical modifications impact the pharmacokinetics/dynamics, off-target effects, and tissue-specific delivery of a NAT. Indeed, chemical modifications play a crucial role in transforming oligonucleotides into pharmaceutical drugs. Multiple rounds of optimization are often required to decipher which chemical modifications, and which combinations of those modifications, should be used in a NAT. Chemical modifications impact metabolic stability, protein binding, toxicity, immunoreactivity, and more [21]. As such, every single parameter must be independently tested following the addition of a chemical modification to assess for changes in pharmacokinetics. For example, both phosphorothioate (PS) backbone modifications and phosphorodiamidate morpholinos (PMO) increase NAT serum stability, yet have opposing actions on plasma protein affinity, with PS increasing affinity and PMOs reducing affinity [21]. With increased plasma protein affinity, NATs will have a longer serum half-life, whereas NATs with reduced plasma protein affinity will be rapidly excreted.

Chemical modifications also impact pharmacodynamics and thus must be tailored for each NAT mechanism: where some modifications enhance gene regulatory effects by improving binding affinity with target RNAs, others can interfere with NAT activity [22]. For example, modifications to ASOs that result in an RNA-like structure will not recruit RNase H1 upon hybridization with target RNA. Hence, RNase H1-dependent ASOs are often synthesized as “gapmer” ASOs composed of a short DNA sequence flanked by heavily modified ribonucleotides to prevent degradation [20]. In contrast, steric block ASOs benefit from adopting modifications that improve stability, regardless of whether they mimic RNA. For siRNAs, both passenger and guide strands are now commonly fully modified. However, the location and type of chemical modification must be tested to ensure proper formation of a functional silencing complex and efficient hybridization with target RNA [10, 22]. Fortunately, our understanding of chemical modifications and their impact on NAT function is rapidly expanding. Indeed, NAT chemistry is an ongoing research area that cannot be fairly summarized here. Recent review articles authored by experts in the field [20–22] provide extensive details regarding advances in nucleic acid chemistry and the modification strategies used for generating successful and clinically approved siRNAs and ASOs. Table 1 describes chemical modifications that are commonly applied to NATs.

**Table 1:** Commonly used nucleic acid modifications for NAT design.

Name	Change in chemistry	Properties
**Backbone modifications**		
Phosphorothioate (PS)	Substitution of one non-bridging oxygen of phosphodiester with a sulfur atom	• Enhanced nuclease resistance
		• Increased lipophilicity
		• Stronger binding to plasma proteins = reduced renal clearance
		• Enhanced cellular uptake
		• Non-specific immune stimulation
		• Reduced binding affinity
Phosphorodiamidate	Substitution of one non-bridging oxygen and one bridging oxygen with NR_2_ groups on phosphodiester linker	• Enhanced nuclease resistance
Peptide nucleic acids (PNA)	Substitution of phosphate backbone with N-2-aminoethylglycine unit	• Increased binding affinity
		• Enhanced nuclease resistance
		• RNase H1 resistant
		• Poor cell permeability
		• Low water solubility
Ethoxy phosphate (P-ethoxy)	Addition of ethyl group to a nonbridging oxygen atom on phosphodiester linker	• Increased lipophilicity
		• Enhanced nuclease resistance
		• Increased binding affinity
Methylphosphonate	Substitution of one non-bridging oxygen with a methyl group	• Increased lipophilicity
		• Enhanced nuclease resistance
		• Increased binding affinity
Phosphorodiamidate morpholinos (PMO)	Substitution of ribofuranose ring with a morpholino ring and a phosphorodiamidate linker replaces the phosphodiester bond	• Reduced binding to plasma proteins
		• Improved tolerability in vivo
		• RNase H1 resistant
Mesylphosphoramidate	Substitution of one non-bridging oxygen with a methanesulfonylamido group	• Reduced toxicity when added to 5′ side of gapmer ASO
		• Enhanced nuclease resistance
**Conjugation**		
PEGylation	Addition of polyethylene glycol (PEG) chain to 3′ or 5′ end of oligonucleotide	• Decreased binding affinity
		• Reduced renal clearance
		• Increased tissue distribution
**Sugar modifications**		
2′-O-methoxyethyl (2′-MOE)	Addition of ethyl group to oxygen at 2′ position on ribose sugar	• Increased binding affinity
		• Enhanced nuclease resistance
		• Decrease immune stimulation
		• Increased half-life
		• RNase H1 resistant
Locked nucleic acid (LNA)	Substitution of hydroxyl group at 2′ position on ribose sugar with covalent linked between 2′ and 4′ positions, forming methylene bridge	• Increased binding affinity
		• Increased risk of hepatotoxicity
		• RNase H1 resistant
2′,4′-constrained ethyl (cET) bicyclic nucleic acids (BNA) (cET-BNAs)	Addition of methyl group to the oxygen on the LNA structure	• Similar characteristics as LNA
		• Reduced hepatotoxicity
2′-O-methylation (2′-OMe)	Addition of methyl group to oxygen at 2′ position on ribose sugar	• Increased binding affinity
		• Enhanced nuclease resistance
		• RNase H1 resistant
2′-Fluoro (2′-F)	Substitution of hydroxyl group at 2′ position on ribose sugar with fluorine atom	• Increased binding affinity
		• Enhanced nuclease resistance
		• RNase H1 resistant
2′-deoxy-2′-fluoro-modified arabinonucleotide (2′-FANA)	Replacement of ribose sugar with 2′-stereoisomer with 2′-F modification	• Increased binding affinity
		• Enhanced nuclease resistance
		• RNase H1 compatible
Unlocked nucleic acid (UNA)	Removal of bond between carbons at the 2′ and 3′ position of the ribose sugar	• Increased flexibility
		• Reduces stability of RNA duplexes
		• Enhanced nuclease resistance when placed at 3′ end
		• RNase H1 resistant

Lack of efficacy is one of the main reasons NATs fail in clinical trials [21]. Two recent examples of failed NATs include drisapersen (an exon skipping ASO developed for Duchenne muscular dystrophy) [23] and tominersen (an ASO targeting the mutated transcript in Huntington’s Disease) [24]. Despite demonstrating promising efficacy in two independent phase II trials, drisapersen development was terminated following its phase III trial as the treatment failed to significantly slow disease progression. Similarly, a phase III trial of tominersen was interrupted due to a lack of observed improvement in treated patients. While phase I and II trials provide valuable insights, phase III clinical trials include a broader patient population with greater genetic diversity. This variation in outcomes between earlier and later stages of clinical development is a common challenge in drug development and requires special consideration when translating promising results from controlled settings to real-world scenarios.

NATs may also fail in clinical trials due to a misunderstanding of their mechanism of action [22]. Off-target effects may mimic the expected mechanism yet remain incapable of eliciting the intended therapeutic benefit in a clinical trial. An example of this is oblimersen (also known as G3139), an 18-nucleotide ASO with a phosphorothioate backbone designed to silence the expression of the anti-apoptotic factor BCL-2 (B-cell lymphoma 2) (ref. [25].). Despite encouraging phase I results, a larger phase III study in older AML patients showed no significant improvement in overall survival [26]. Subsequent investigations revealed that oblimersen’s efficacy was not due to selective BCL-2 downregulation, but rather non-specific apoptosis-related off-target effects induced by its phosphorothioate backbone [27]. To mitigate this, we suggest detailed mechanistic studies to help ensure clinical success.

Finally, an ongoing challenge in the clinical applicability of NATs depends on efficient and specific delivery to extrahepatic tissues. Due to their large size, anionic charge, and susceptibility to nucleases, many NATs require carriers for cellular entry. Lipid-carriers, such as liposomal nanoparticles (LNPs), are commonly used for the systemic delivery of NATs [28]. However, approximately 90% of intravenously injected LNPs localize to the liver [29], meaning other organ systems receive only a fraction of the injected NAT. Furthermore, delivery entails not only transporting NATs to the target cells (i.e., cellular uptake and biodistribution), but also ensuring sufficient internalization within those cells. Even when LNPs reach the target cells, endosomal escape is limited, with only 2–3% of packaged nucleic acids reaching the cytosol [30]. Fortunately, improving endosomal escape and tissue-specific targeting are major ongoing research areas in the field (reviewed here [31, 32]).

### Myeloid leukemia-specific challenges in NAT development

AML presents unique challenges for NAT development. While cancer-specific surface markers have propelled the development of immunotherapies, there are limited tumour-associated antigens that are unique to AML blasts [33]. Indeed, AML proves to be particularly challenging to selectively target because both leukemic blasts and healthy cells tend to express the same antigen markers. As a result, clinical trials using targeted immunotherapies in AML have failed due to significant toxicities [34], and similar challenges apply when designing targeted NATs for AML. In addition to universal delivery challenges of NATs, it remains challenging to deliver to myeloid malignancies due to the slow vasculature flow and highly mineralized extracellular matrix of the bone marrow [35]. Therefore, bone marrow-delivery may require higher drug doses to overcome the low bioavailability and achieve therapeutic levels.

Most notably, the heterogenous nature of AML and the lack of preclinical models that can recapitulate the intra- and interpatient heterogeneity has resulted in the notoriously slow development of targeted therapies for AML [36]. The current knowledge of clonal evolution of AML suggests that it is the culmination of many mutations that result in the selection and growth of leukemic clones [37]. Therefore, therapies that target only one or two driver mutations of the disease are likely not effective to rid all leukemic cells, and/or may cause selection pressure and the emergence of subclones, leading to disease relapse and resistance. Consequently, NATs must be developed with this heterogenous nature in mind, likely including synergistic combination therapies. As such, testing and developing a new NAT for AML includes additional pre-clinical studies to determine the best drug combinations and doses for efficacy and safety. Furthermore, clinical trial design for NATs must consider patient stratification strategies to favour improved outcome, ensuring the right NATs are given for the right subset of AML patients. Stratification may include the clinical transcriptome-based assay recently described for AML risk stratification [38] but getting sufficient enrollment for the particular subtype can take additional time. Nonetheless, given that myeloid leukemia patients often lack therapeutic options, there is a pressing need for NATs even if only for a small subset of patients.

## Application of nucleic acid therapeutics to myeloid malignancies

We categorized recent reports exploring the application of NATs to myeloid leukemias into five target classes: fusion oncogenes, signal transducers involved in programmed cell death, cell division and/or growth, non-coding RNA, transcription factors, and cell surface receptors. Within each of these categories, NATs targeting a specific gene were grouped into a subcategory. We describe the rationale for targeting that gene in myeloid malignancies, the experimental design and results reported in each study, and provide insight into potential future directions.

### Fusion oncogenes

#### RUNX1::ETO

Approximately 10% of AML cases are caused by the t(8;21) translocation resulting in the *RUNX1::ETO* gene product that inhibits granulocytic differentiation and promotes excessive proliferation [39]. RUNX1::ETO is reported to act as a transcriptional repressor by recruiting histone deacetylases and DNA methyl transferases to tumour suppressor genes and critical myeloid transcription factors [40].

Recently, RUNX1::ETO was inhibited using an siRNA targeting the fusion site, which resulted in myeloid differentiation in vitro and in vivo and increased overall survival in xenograft murine models of AML [41]. RUNX1::ETO siRNA encapsulated in LNPs labeled with an in vivo-compatible dye were intravenously injected into Kasumi-1 AML-engrafted mice, confirming LNPs uptake in leukemic cells and accumulation in leukemic reservoirs. RUNX1::ETO siRNA-treated mice exhibited efficient knockdown of RUNX1::ETO expression, reduced growth of leukemic cells in vivo, and improved survival compared to control siRNA. Importantly, when untreated mice were re-transplanted with leukemic cells isolated from previously treated mice, engraftment was markedly reduced with 50% of secondary recipient mice never developing AML. Previous RUNX1::ETO siRNA studies demonstrated that RUNX1::ETO depletion induces terminal differentiation [42, 43]. Taken together, these results indicate that targeting RUNX1::ETO via siRNA could reduce the aggressivity and proliferative capacity of leukemic cells. Nonetheless, further studies are needed to assess the curative potential of siRNA-mediated inhibition of RUNX1::ETO combined with currently used therapies such as hypomethylating agents.

#### BCR::ABL1

The *BCR::ABL1* oncogene results from the translocation of chromosome 9 and chromosome 22, also termed the “Philadelphia chromosome”, found in patients with chronic myeloid leukemia (CML). *BCR::ABL1* encodes the BCR::ABL1 oncoprotein with constitutive tyrosine kinase activity that leads to uncontrolled proliferation of myeloid progenitor cells that, unlike AML, can still terminally differentiate [44]. Small molecule tyrosine kinase inhibitors, the first of which was imatinib, have been developed to inhibit BCR::ABL1 activity. However, resistance via point mutations in the BCR::ABL1 protein remains a clinical problem. Furthermore, many studies have evaluated NATs to target BCR::ABL1 due to its well-established importance in disease initiation. In addition, experimental models of CML, such as the cell line K562 isolated from a patient in blast crisis, exhibit very similar characteristics to AML, making them useful for modeling the disease and understanding the application of NATs in AML.

The first siRNA designed against the *BCR::ABL1* fusion mRNA was reported by Wilda et al. [45]. By transfecting a *BCR::ABL1* fusion-targeting siRNA into K562 cells, mRNA and protein levels were downregulated resulting in morphological changes indicating megakaryocytic differentiation compared to controls. While silencing potential was well-established, the authors failed to improve the siRNA design with chemical modifications. Nonetheless, this study introduced the possibility of developing targeted RNA-based therapies that selectively act on tumor-specific fusion mRNAs while sparing normal cells. As such, many other studies investigating *BCR::ABL1* siRNAs [46, 47] and ASOs [48] have been explored.

Sixteen years later, Valencia-Serna et al. [49]. demonstrated in vivo administration of a functional *BCR::ABL1* siRNA effectively silenced *BCR::ABL1* mRNA expression and suppressed the growth of a xenotransplanted CML tumour in nude mice. To address the emergence of BCR::ABL1 mutations, such BCR::ABL1 targeted NATs could be useful for patients exhibiting resistance to BCR::ABL1 small molecule inhibitors, and to further understand the long-term potential of using NATs to target fusion oncogenes.

### Signal transducers involved in programmed cell death, cell division and/or growth

#### BCL-2

BCL-2 is often overexpressed in AML blasts and is associated with a poor prognosis and chemotherapy resistance. Furthermore, a murine leukemia model with conditional expression of BCL-2 demonstrated that leukemic cells depend on BCL-2 for survival and growth [50]. As such, inhibiting or downregulating BCL-2 is a logical treatment strategy. Venetoclax, a small molecule BH3-mimic, inhibits BCL-2, forcing cells into programmed cell death and is prescribed for elderly, newly diagnosed or relapsed/refractory AML. While targeted BCL-2 inhibition with venetoclax in combination with hypomethylating agents or low-dose cytarabine can achieve clinically meaningful responses in up to 70% of patients, the acquisition of resistance to therapy frequently occurs within a median time of approximately 1 year, through mechanisms such as overexpression of other BCL-2-family members [51]. In this context, NATs could provide complementary therapeutic strategies to target emerging mechanisms of resistance.

First designed and tested for leukemia in 1990 [25], BCL-2-targeting ASOs are arguably the most extensively studied NATs in myeloid leukemias. The next generation BCL-2 ASO, BP1002, has improved pharmacokinetic properties compared to the first-generation BCL-2 ASO, oblimersen. BP1002 is incorporated into a neutral-charge, non-toxic liposome and contains a fully p-ethoxy modified backbone, a modification that masks the internucleotide phosphate charge with an ethyl group, improving the oligonucleotide’s nuclease resistant and limiting off-target effects [52]. BP1002 is particularly relevant for venetoclax-resistant leukemic cells, which have 2-3-fold higher BCL-2 expression than sensitive cells. Treating such cells with BP1002 in combination with decitabine resulted in ~60% decrease in cell proliferation compared to venetoclax with decitabine [53]. The phase I trial of BP1002, which is now recruiting (end date August 2024, NCT05190471) will assess BP1002 as a monotherapy for relapsed AML and BP1002 with decitabine for refractory AML. Further research could explore the combination of BP1002 with novel anti-apoptotic inhibitors [54] to address venetoclax-resistance.

#### MCL-1

Programmed cell death is often dysregulated in myeloid malignancies due to the upregulation of anti-apoptotic factors such as BCL-2 and MCL-1 (myeloid cell leukemia sequence 1). These proteins inhibit apoptosis via protein-protein interactions with pro-apoptotic proteins BAX, BAK and BH3-only proteins, thereby preventing the formation of the mitochondrial outer membrane pore and release of cytochrome C [55]. In addition to the role of MCL-1 in the regulation of apoptosis, MCL-1 is involved in controlling the myeloblast to neutrophil transition [56]. Historically, inhibiting MCL-1 has proven challenging due to the lack of specificity, poor cell permeability, and weak binding by small molecule inhibitors [57].

With the goal of inhibiting MCL-1 using NATs, Yang et al. [58]. demonstrated the differentiation-inducing effects of siRNA-mediated knockdown of MCL-1 in AML cell lines. MCL-1-targeting siRNA or control siRNA were electroporated into ATRA-sensitive NB4 and PL21 cell lines, inducing >10-fold protein knockdown. MCL-1 silencing in NB4 and PL21 cells increased myelomonocytic differentiation as measured by CD11b. As MCL-1 downregulation contributes to ATRA-mediated differentiation, the authors hypothesized that further MCL-1 downregulation could potentiate ATRA’s effects. Indeed, co-treatment of NB4 and PL21 cells with 0.1 μM ATRA and MCL-1 siRNA resulted in 2.5-fold increase in C/EBPε (regulator of promyelocyte to myelocyte transition) expression compared to cells treated with ATRA and control siRNA. Furthermore, MCL-1 siRNA treatment sensitized the ATRA-resistant cells U937 and KG-1 to ATRA-mediated differentiation, resulting in an increase of CD11b expression compared to controls. This finding was supported in another study demonstrating MCL-1 siRNA mediated inhibition of leukemic cell proliferation and viability [59].

The results described to-date open new combination therapy possibilities. For example, MCL-1 siRNA with the BCL-2 inhibitor venetoclax could provide a synergistic effect as both cooperate on the same pathway. Furthermore, as constitutive FLT3 signalling due to FLT3-ITD mutations enhances MCL-1 expression [60], combining FLT3 inhibitors with MCL-1 siRNA could potentiate the effects of inhibiting downstream FLT3 signalling. Despite these possibilities, these combination therapies should be tested with caution as MCL-1 is expressed endogenously in all cells and regulates the critical process of cell division/cell death. Therefore, selective delivery and tight regulation of the dose would be necessary to direct MCL-1 inhibition to high MCL-1-expressing malignant cells.

#### Grb2

Grb2 (growth factor receptor-bound protein 2) is a critical component of tyrosine kinase signalling. With over 50% of AML patients having tyrosine kinase-related mutations (i.e., FLT3-ITD, c-kit, JAK2) that require enhanced Grb2 activity to drive oncogenic pathways [61], inhibiting Grb2 is an interesting therapeutic target.

First reported by Tari et al. [62]. in 1999, BP-1001 is an 18-nucleotide ASO with p-ethoxy backbone incorporated into a neutral liposome. The neutral nature of the liposome reduces interactions with plasma proteins, increasing both circulation time and cellular uptake. Preclinical studies of BP-1001 in a murine xenograft model of BCR::ABL1-positive CML cells demonstrated that treated mice had a 2-fold increase in overall survival compared to mice receiving a control liposome [63]. This prompted the first phase I clinical trial of BP-1001 in refractory/relapsed AML and CML patients [64]. No dose-related toxicity was noted for BP-1001 and only one of 21 patients in the trial experienced BP-1001-related toxicity. The superior safety profile of BP-1001 was attributed to the p-ethoxy backbone modification which does not induce complement activation or coagulation. Five of six patients treated with only one cycle of BP-1001 plus low-dose cytarabine had a reduction in bone marrow blasts (>50% reduction), and three out of the six achieved complete remission (with or without incomplete haematological recovery). Together these results demonstrate BP-1001’s anti-leukemic activity for relapsed/refractory AML. Currently, a phase IIa trial of BP-1001 in combination with venetoclax and decitabine in untreated and refractory/relapsed AML (NCT02781883) is underway, with an estimated completion date of December 2024.

### Non-coding RNA

#### miR-21 & miR-196b

Over 50% of AMLs exhibit *HOXA9* (Homeobox A9) overexpression often caused by mixed-lineage leukemia (MLL)-translocations or *NPM1* (Nucleophosmin1) mutations. MLL fusion proteins interact with other HOX co-activators and preferentially activate *HOX* gene transcription. In normal hematopoiesis, HOXA9 maintains the proliferative ability and clonogenicity of progenitor cells in part by maintaining the expression of microRNAs miR-21 and miR-196b. As such, many AML patients present with increased levels of miR-21 and miR-196b [65].

To counteract the pro-leukemic effects of *HOXA9* overexpression, Velu et al. [65]. designed antagomirs against miR-21 and miR-196b. The antagomiR-21 (a21) and antagomiR-196b (a196b) sequences are perfectly complementary to their respective targets. The antagomirs contain phosphorothioate linkages and 2′-OMe sugars to increase stability and affinity, and a 3′ cholesterol modification to interact with lipoprotein receptors on myeloid lineage cells. After confirming miRNA knockdown, the impact of a21 and a196b on proliferation was assessed in MLL-fusion protein-expressing cells; both their combined and individual use decreased colony-forming units, indicating specific reduction in oncogenic HOX-related proliferation. Next, the in vivo therapeutic potential of a21 and a196b was tested in combination with a standard cytarabine and anthracycline regimen. Immunodeficient mice (NOD/SCID/SGM3 mice) were implanted with primary human hematopoietic cells transformed with both MLL-AF9 fusion protein and oncogenic NRAS. Following engraftment, the mice were treated with a consistent release of antagomirs or controls via a pump for 6 weeks. Mice treated with a21 + a196b and induction chemotherapy (cytarabine/doxorubicin) survived longer than mice treated with controls and chemotherapy, demonstrating the powerful anti-leukemic effect of antagomir therapy, especially in combination treatments. Though there are currently no co-delivery systems for NATs, this study demonstrates the synergistic potential of co-developing NATs that modulate different targets from the same pathway.

#### miR-181a

microRNA 181a (miR-181a) is downregulated in specific leukemias, particularly CML. Previous reports demonstrate the anti-proliferative activity of miR-181a mimics in the CML cell line K562 [66]. However, short, pre-processed miRNA mimics cannot fully recapitulate the endogenous primitive (pri)-miRNA structure that may have unknown, but physiologically important, actions in the cells. Therefore, Su et al. [67]. developed saRNA targeting the miR-181a promoter, “saRNA-3”, that induced a 2.5-fold increase in relative pri-miR-181a expression and inhibited proliferation and colony forming capacity of the cells. To assess the potential imatinib-sensitizing effect of increased miR181a expression, saRNA-3 or control RNA were administered to immunodeficient mice engrafted with imatinib-sensitive or imatinib-resistant K562-luciferase cells. Co-administration of saRNA-3 with imatinib decreased leukemic burden (indirectly measured by luciferase bioluminescence) and increased overall survival compared to control saRNA, in both imatinib-sensitive and -resistant cohorts. Future research could investigate the differential effects of miRNA mimics and saRNAs on miR-181a’s anti-leukemic action, aiding in classifying the various ncRNA-targeting approaches.

### Transcription factors

#### STAT3

Signal transduction and activator of transcription (STAT) proteins are a family of transcription factors frequently overactive in various cancer types, leading to the upregulation of genes involved in cell proliferation, anti-apoptosis, angiogenesis, and immune evasion. In the context of myeloid leukemias, the significance of targeting STAT3 stems from its control over immune cell differentiation and proliferation. STAT3 is overexpressed in leukemic stem cells and associated with poor prognosis [68], remaining an important target for NAT development.

As STAT3 has been established as a key player in many types of cancer, a novel STAT3 ASO, known as AZD9150, is currently undergoing clinical investigation for the treatment of lymphoma and lung cancer [69]. AZD9150 is 16-nucleotide ASO containing 10 phosphorothioate modified DNA nucleotides flanked by three constrained ethyl-bridged nucleic acid (cET-BNA) residues (described in Table 1) on both ends. STAT3 gene expression is reduced due to the binding of the ASO to the 3′-UTR of *STAT3* mRNA, preventing proper translation. AZD9150 treatment caused knockdown of STAT3 expression in AML cell lines and human primary myelodysplastic syndrome (MDS)/AML stem cells [68], resulting in an increase in erythroid and myeloid differentiated colonies. Importantly, no such change in differentiated colonies was noted in AZD9150-treated samples from healthy controls. Next, to assess the translatability of these in vitro and ex vivo experiments, AZD9150 was administered to irradiated immunodeficient mice transplanted with AML bone marrow-derived mononuclear cells. Compared to non-targeting control ASO, AZD9150 treatment decreased malignant cells engraftment in the bone marrow. AZD9150 treatment also induced erythroid and myeloid differentiation in MDS stem cell colonies. A common symptom of MDS is cytopenia due to inefficient hematopoiesis. Therefore, using a differentiation-inducing therapy like AZD9150 could provide patients with MDS an opportunity to increase red blood cell, neutrophil, and/or platelet count without the high burden of regular blood transfusions.

An alternative form of STAT3 inhibition was described using siRNA conjugated to a cytosine-phosphate-guanine (CpG) oligonucleotide for selective targeting to cells expressing toll-like receptor 9 (TLR9). TLR9 endogenously recognizes and engulfs bacterial and viral DNA containing CpG motifs, thereby CpG-siRNA conjugates were delivered preferentially to TLR9-positive cells. As TLR9 is highly expressed on myeloid-derived cells, this is of particular interest for myeloid malignancies. Hossain et al. [70]. demonstrated targeted delivery of CpG-siRNA to murine leukemic cells and subsequent STAT3 knockdown in vivo resulting in increased percent survival and reduced leukemic cell penetrance in bone marrow and spleen. The authors further demonstrated that reduction in leukemic burden was attributed to the CpG-STAT3 siRNA-induced differentiation of leukemic cells to antigen-presenting cells expressing immunostimulatory markers, ultimately recruiting CD8 + T-cells. With limited immunotherapy options for AML, this study demonstrates the potential of NAT-mediated differentiation to sensitize the immune system to leukemic cells. Further testing in humanized AML mouse models would provide stronger evidence for the siRNA as an immunotherapy. A challenge with studying the role of the immune system in leukemia progression is that AML xenograft models cannot recapitulate the immune phenotype observed in immunocompetent mice and humans because engraftment of human AML is not possible without T-cells, B-cells, and NK cells ablation [71].

#### DDX5

DEAD-box RNA helicase (DDX5) is a multiprotein complex that controls the expression of many transcription factors. AML cells demonstrate dependence on DDX5 to maintain their proliferative capability [72]. Therefore, Wu et al. [73]. designed and validated the therapeutic potential of DDX5-targeting siRNA in APL cell lines NB4 and HL-60. DDX5 protein knockdown in NB4 and HL-60 cells resulted in a decrease in proliferating cells and an increase in CD14-expressing cells compared to control siRNA-treated cells, suggesting that DDX5-siRNA treatment induced monocytic differentiation in both cell lines. As the focus of this study was not to develop a therapeutically relevant NAT targeting DDX5 for AML, further work is necessary to validate the in vivo translatability. Furthermore, potential repurposing of DDX5-targeting phosphorothioate-modified ASO [74] (originally designed for treatment prostate cancer) may be useful for AML.

### Cell surface receptors

#### CD33

CD33 is a myeloid differentiation antigen that is expressed on myeloid progenitor cells, such as myeloblasts or monoblasts, but is not expressed on multipotent progenitor cells. As AML blasts have elevated expression of CD33, it is a potential target [75]. As such, gemtuzumab ozogamicin, a CD33-targeted monoclonal antibody conjugated to the cytotoxic agent ozogamicin was approved by the FDA as a targeted therapy for AML. Antibodies often elicit immune responses in patients, therefore, aptamers may be advantageous in terms of immune response [76].

Yang et al. [77]. therefore developed a CD33-specific DNA aptamer that targets CD33-positive cells and differentiated between CD33-expressing and non-expressing cells in vitro and in vivo. The aptamer was identified using cell-SELEX with positive and negative screening. The CD33-aptamer was assessed for in vivo biodistribution and specificity using immunodeficient mice with CD33-positive (HL-60) or CD33-negative (A549) tumors. Labeled CD33-aptamer localized to CD33-positive tumors, liver, and kidneys within 1 hour, sustaining this localization even after 24 h. No aptamer was found in CD33-negative tumors, confirming its targeting ability. A shorter but functional version of the aptamer was then loaded with the anthracycline doxorubicin. Targeted delivery of doxorubicin could limit the off-target consequences while increasing the cytotoxic payload reaching leukemic cells. Treatment of CD33-positive HL-60 cells with doxorubicin-aptamer conjugate resulted in cell cycle arrest in G2 phase, whereas no such effect was noted with CD33-negative cells.

In addition to CD33, many aptamers are being developed for other AML targets including nucleolin [78], CD123 (ref. [79].), and CD117 (ref. [80].), yet very few have been tested for clinical application. While aptamers have documented advantages over antibodies in terms of size and production, a head-to-head comparison of the CD33-targeted aptamer compared to the clinically approved CD33 antibody gemtuzumab would be useful for understanding the limitations and opportunities for aptamer based in vivo targeting.

## Future opportunities for differentiation-inducing NATs in myeloid malignancies

Here we discuss future opportunities for differentiation-inducing NATs in myeloid leukemias, highlighting potential targets identified from fundamental mechanistic studies and discussing possible solutions for improving delivery to leukemic cells.

### Potential NAT targets identified from fundamental biology studies

The genetic heterogeneity of myeloid leukemias is associated with treatment challenges, yet it also opens the door for personalized treatment approaches with NATs. Many dysregulated factors have been identified; in particular, transcription factors and ncRNAs that influence differentiation but are challenging to target via small molecules and proteins. For example, the transcription factor targets *MYB* and *GFI1* were identified in multiple screens using RNAi [81] and CRISPR [82, 83] where loss of function favored myeloid differentiation. Furthermore, transcription factors that govern healthy myeloid differentiation (reviewed [5]) can also be fine-tuned using NATs to reprogram leukemic cells to a healthy phenotype. Similarly, numerous ncRNAs including lncRNAs [84], miRNAs [85–87], and circRNAs [88] have been identified via RNA-sequencing, RT-qPCR, and microarray experiments in AML. These targets can be blocked using antagomirs, mimicked using siRNAs/saRNAs, or increased via saRNAs to promote differentiation (Table 2).

**Table 2:** Examples of experimentally identified targets in myeloid leukemias that would benefit from a nucleic acid therapeutic approach.

Target	Relevance to myeloid leukemia	Potential NAT Strategy	Ref.
**Transcription factors**			
*MYB*	• Downregulated in response to differentiation-inducing agent. • Knockdown of each gene resulted in similar transcriptional motifs as in PMA-treated cells.	siRNA, ASO	[81]
*HOXA9*			
*CEBPG*			
*GFI1*			
*CEBPA*			
*FLI1*			
*MLLT3*			
*ZFP36L2*	• Knockdown of gene resulted in increased expression of CD14 and/or CD11b, indicators of myeloid differentiation. • Identified in a genome-wide loss of function CRISPR/Cas9 screen in THP-1 cells.	siRNA, ASO	[82]
*DOT1L*			
*HDAC3*			
*KDM1A*			
*MED12*			
*PRMT1*			
*STK11*			
*EP300*			
*MED16*			
*MED24*			
*KAT2A*	• Downregulation of *KAT2A* induced myeloid differentiation and decreased proliferation in AML cell lines and primary AML blasts. • KAT2A was identified through a CRISPR dropout screen.	siRNA, ASO	[83]
**Non-coding RNAs**			
miR-10a/b	• Overexpressed in AML subtypes (NPM1-mutated, t(8;21), t(9;11)). • Expression of miR-10a/b gradually decreases through granulocytic and monocytic differentiation. • Overexpression of miR-10a resulted in increased AML cell proliferation and inhibited ATRA-induced granulocytic differentiation.	siRNA, ASO	[85]
miR-29a/b/c	• Downregulated in AML BM blasts compared to healthy donors. • Reintroduction of miR-29a/b/c corrected myeloid differentiation arrest in vitro and reduced leukemic burden in vivo.	saRNA, miRNA mimic	[87]

### Improving delivery of NATs with nucleic acid conjugates

There is ongoing work to improve NAT delivery to leukemic cells including the use of red-blood cell derived extracellular vesicles [89], lipid-polymer nanoparticles [90], and DNA origami nanostructures [91]. To further improve delivery, more efforts to conjugate delivery vehicles with aptamers could be explored. Indeed, SELEX enables aptamers to differentiate leukemic cell-types [92] and potentially target leukemic cells over healthy cells. New aptamers could be selected for AML-specific targets such as CLL1, CD99, CD157, and TIM3 [93–95]. Furthermore, given the clonal heterogeneity of AML, future efforts should examine whether decorating a delivery vehicle with a combination of aptamers targeting different cell surface markers improves outcomes in this heterogenous disease.

### How to design your own NAT

**Fig. 5:**
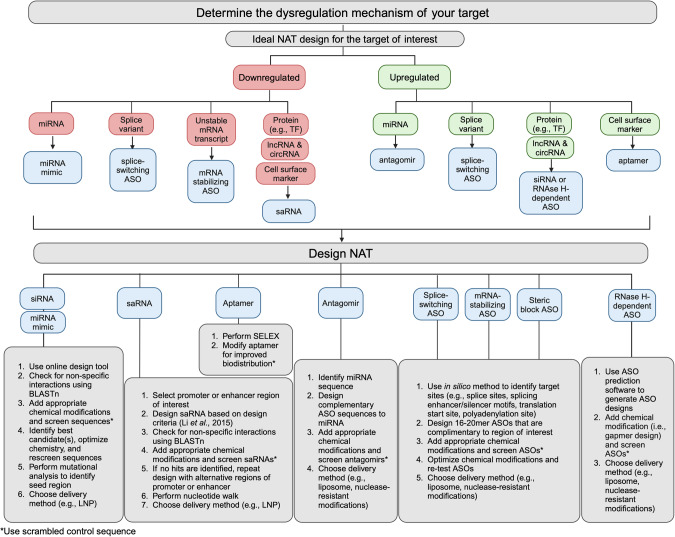
Identification and design of NATs for myeloid leukemia targets. This chart provides a step-by-step guide to identifying the ideal NAT for your target and then designing and screening that NAT.

“Drugging the genome” is now a reality thanks to the major developments in NATs over the past decades. Designing a NAT starts with identifying the target sequence and deciphering the target dysregulation mechanism. Next, the class of NAT is selected based on target expression and dysregulation. The literature contains design outlines for each type of NAT (siRNA [96], ASO [97], saRNA [98], aptamer [99]); however, design rules are improved each year. Figure 5 provides a guide to identify and design the best-suited NAT for your target.

Finding a lead NAT requires designing, synthesizing, and screening hundreds to thousands of sequences. In silico tools allow for the prediction of certain off-target effects, but so far, no tool has reliably predicted toxicity based on chemical modifications. Therefore, NAT design must be a collaborative effort with oligonucleotide experts and leukemia researchers to maximize therapeutic effect and minimize toxicity. Specific safety considerations that are unique to NATs are reviewed here [100].

In an academic setting, high-throughput screening may not always be feasible, therefore performing iterative rounds of small-scale screening assays may be necessary. Upon proof-of-concept, additional pre-clinical studies may require collaboration with large institutions or companies to validate and optimize the hit.

### Concluding remarks

Drug development for myeloid leukemias lags behind that of many other cancers, resulting in poor treatment options for patients with these malignancies. NATs exhibit versatility in their capacity to target and modulate specific genetic and molecular aberrations that underlie myeloid leukemias, offering a promising avenue for tailored and effective treatments. As our understanding of myeloid diseases deepens, and therapeutic strategies continue to evolve, NATs are poised to play a vital role in improving outcomes and quality of life for patients with myeloid leukemias.
